# Autophagy Inhibition Enhances Apoptosis Induced by Dioscin in Huh7 Cells

**DOI:** 10.1155/2012/134512

**Published:** 2012-11-05

**Authors:** Ming-Ju Hsieh, Shun-Fa Yang, Yih-Shou Hsieh, Tzy-Yen Chen, Hui-Ling Chiou

**Affiliations:** ^1^School of Medical Laboratory and Biotechnology, Chung Shan Medical University, 110, Section 1, Chien-Kuo N. Road, Taichung 402, Taiwan; ^2^Institute of Medicine, Chung Shan Medical University, 110, Section 1, Chien-Kuo N. Road, Taichung 402, Taiwan; ^3^Department of Medical Research, Chung Shan Medical University Hospital, 110, Section 1, Chien-Kuo N. Road, Taichung 402, Taiwan; ^4^Department of Biochemistry and Institute of Biochemistry and Biotechnology, Chung Shan Medical University, 110, Section 1, Chien-Kuo N. Road, Taichung 402, Taiwan; ^5^Department of Internal Medicine, Chung Shan Medical University Hospital, 110, Section 1, Chien-Kuo N. Road, Taichung 402, Taiwan; ^6^Department of Internal Medicine, School of Medicine, Chung Shan Medical University, 110, Section 1, Chien-Kuo N. Road, Taichung 402, Taiwan; ^7^Department of Clinical Laboratory, Chung Shan Medical University Hospital, 110, Section 1, Chien-Kuo N. Road, Taichung 402, Taiwan

## Abstract

Extensive research results support the application of herbal medicine or natural food as an augment during therapy for various cancers. However, the effect of dioscin on tumor cells autophagy has not been clearly clarified. In this study, the unique effects of dioscin on autophagy of hepatoma cells were investigated. Results found that dioscin induced caspase-3- and -9-dependent cell apoptosis in a dose-dependent manner. Moreover, inhibition of ERK1/2 phosphorylation significantly abolished the dioscin-induced apoptosis. In addition, dioscin triggered cell autophagy in early stages. With autophagy inhibitors to hinder the autophagy process, dioscin-induced cell apoptosis was significantly enhanced. An inhibition of caspase activation did not affect the dioscin-induced LC3-II protein expression. Based on the results, we believed that while apoptosis was blocked, dioscin-induced autophagy process also diminished in Huh7 cells. In conclusion, this study indicates that dioscin causes autophagy in Huh7 cells and suggests that dioscin has a cytoprotective effect.

## 1. Introduction

In recent years, products derived from natural plants have been gaining more and more attention for the intervention of malignant invasive progression in the late stage of neoplastic diseases [1] or as potent chemopreventive drugs [2], especially for relatively chemorefractory tumors such as hepatocellular carcinoma (HCC) [3].

**Figure 1:**
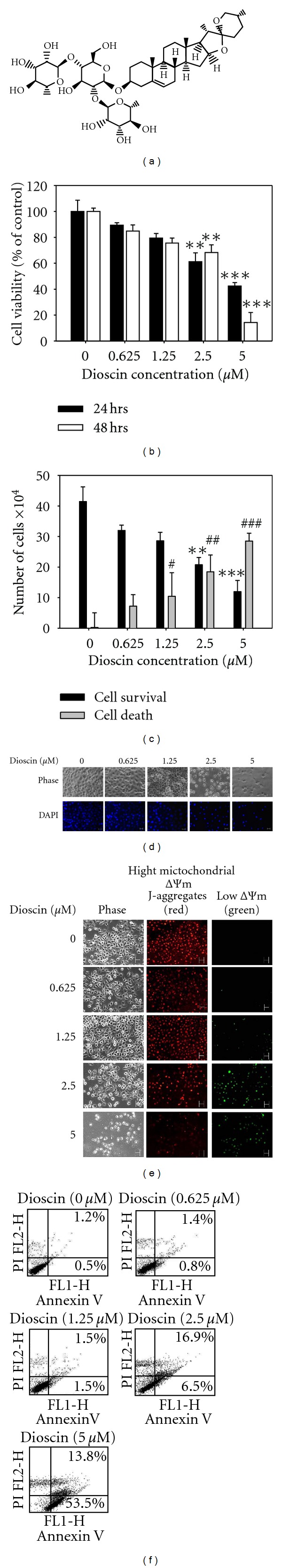
Dioscin exerts apoptotic effect on Huh7 cells. (a) Structure of dioscin. (b) Cell viability of Huh7 cells cultured in presence of dioscin for 24 and 48 hours, as analyzed by MTT assay. (c) Cell survival and cell death, determined by cell count, of Huh7 cell treated with dioscin for 24 hours. Cells were treated with an indicated concentration of dioscin for 24 hours and then subjected to DAPI staining (d) and JC-1 (e) followed by an observation under fluorescence microscopy. For quantitative analysis of apoptosis, dioscin-treated cells were harvested and then subjected to Annexin-V and PI double-stained flow cytometry (f). Results are shown as mean ± SD from 3 determinations per condition repeated 3 times. **P < 0.01; ***P < 0.001, compared with the control (0 *μ*M); ^#^ *P* < 0.05; ^##^ *P* < 0.01; ^###^ *P* < 0.001, compared with the control (0 *μ*M). Scale bars = 100 *μ*m.

Previous studies have indicated that *Dioscorea nipponica extract *(DNE) could reduce the metastasis of melanoma in vitro and in vivo through inhibited phosphorylation of Akt, activation of NF-*κ*B, and increase the expression of I*κ*B [4]. Furthermore, Du et al. have isolated and identified a new water-soluble steroidal saponin from *Dioscorea nipponica* Makino and defined its chemical structure (as shown in Figure 1(a)) [5]. This newly isolated plant steroidal saponin is named dioscin. The diosgenyl saponin dioscin is one of the most common steroidal saponins found in plants and exhibits cytotoxicity in several cancer cells. It has been extensively studied on its antitumor effect by antiproliferative activities, cell cycle arrest, and induced apoptosis via the mitochondrial and some other pathway [6–9]. Results indicated that dioscin is able to induce Hela cells apoptosis via the inhibition of Bcl-2 and activation of caspases-9 and caspase-3 [10] and cause generation of reactive oxygen species (ROS) in HL-60 cells to induce apoptosis [11]. Furthermore, its capability to decrease the resistance degree of HepG2/adriamycin cells via a significant inhibition of P-glycoprotein expression has been proven and, therefore, was proposed to be a potent multidrug resistance reversal agent [12]. However, the effect of dioscin on tumor cells autophagy has not been clearly clarified.

Autophagy is a major intracellular degradation mechanism operating under stress conditions to promote survival during starvation or lead to programmed cell death type II under specific conditions such as the inhibition of apoptosis [13–15]. The process of autophagy is initiated by engulfing large sections of cytoplasm by a crescent-shaped phagophore that elongates to autophagosome, which subsequently fused with a lysosome and its contents are degraded by lysosomal hydrolases [16–18]. Since autophagy is vital in regulating growth and maintaining homeostasis in multicellular organisms, defective autophagy contributes to pathogenesis of a number of diseases, including myopathies, neurodegenerative diseases, and some forms of cancers [19]. The aim of this study was to characterize the effects of dioscin and underlying molecular mechanism on autophagy and apoptosis in dioscin-induced cytotoxicity.

## 2. Materials and Methods

### 2.1. Chemicals

Dioscin of ≥98% purity was purchased from China Langchem INC. (St. Caliun, Shanghai). Stock solution of dioscin was made at 10 mM concentration in dimethyl sulfoxide (DMSO) (Sigma, St. Louis Co.) and stored at −20°C. The final concentration of DMSO for all treatments was less than 0.1%. Other chemicals, including 3-(4,5-dimethylthiazol-2-y1)-2,5-diphenyltetrazolium bromide (MTT), paraformaldehyde, Triton X-100, bafilomycin A1 (BafA1), 4′-6-Diamidino-2-phenylindole (DAPI), 3-Methyladenine (3-MA), p38 MAPK inhibitor SB203580, and JNK1/2 inhibitor SP600125 were obtained from Sigma Chemical Co. (St. Louis, MO, USA). The ERK1/2 inhibitor U0126 and general caspase inhibitor Z-VAD-FMK were purchased from Promega (Madison, WI, USA). Specific caspase inhibitors for caspase 3 (Z-DEVE-FMK), caspase 8 (Z-IETD-FMK), or caspase 9 (Z-LEHO-FMK) were purchased from BioVision (Mountain View, CA). NE-PER Nuclear and Cytoplasmic Extraction Kit and BCA protein assay reagent were purchased from Thermo. The FITC Annexin V Apoptosis Detection Kit I was obtained from BD Biosciences, USA. Antibodies for Beclin-1, LC3, cleaved PARP, caspase-8, caspase-9, and Bcl-2 were obtained from Cell Signaling; antibodies for cytochrome c and caspase 3 were obtained from Invitrogen, CA; antibodies for p38, JNK1/2, and *β*-actin were obtained from BD Biosciences, USA; antibodies for ERK1/2, p-ERK1/2, p-p38, and p-JNK were obtained from Millipore Corporation, Milford, MA, USA.

### 2.2. Cell Culture

Huh-7, a human hepatocellular carcinoma cell line, obtained from the Food Industry Research and Development Institute (Hsinchu, Taiwan), was cultured in Dulbecco's modified Eagle's medium (DMEM) (Gibco BRL, Grand Island, NY, USA) supplemented with 10% fetal calf serum (FCS), 1 mM glutamine, 1% penicillin/streptomycin, 1.5 g/L sodium bicarbonate, and 1 mM sodium pyruvate (Sigma, St. Louis, Mo, USA). The cell cultures were maintained at 37°C in a humidified atmosphere of 5% CO_2_.

### 2.3. Cell Cytotoxicity and Cell Count Assay

The effect of dioscin on cell growth was assayed by the MTT method, as previously described [4]. Briefly, 2 × 10^5^ cells/well were cultured in 6-well plates and stimulated with different concentrations of dioscin (0, 0.625, 1.25, 2.5, 5 *μ*M). After 24 or 48 hours, MTT was added to each well (at a final concentration of 0.5 mg/mL) and incubated for further 4 hours. The viable cell number was directly proportional to the production of formazan, reflected by the color intensity measured at 570 nm, following the solubilization with isopropanol. Cell proliferation was also evaluated by a cell count assay. Briefly, 2 × 10^5^ cells/well were cultured in 6-well plates and stimulated with different concentrations of dioscin (0, 0.625, 1.25, 2.5, 5 *μ*M). After 24 or 48 hours, the cells were trypsinized and centrifuged, viable and death cells were counted using a hemocytometer after staining with trypan blue. Each condition was performed in 3 replicate wells and data were obtained from at least 3 separate experiments. 

### 2.4. Preparation of Cell Nuclear and Cytosolic Extracts

Nuclear extracts and cytosolic extracts were prepared essentially as described [20]. NE-PER nuclear and cytoplasmic extraction reagents were used to prepare extracts. Briefly, cells were washed with cold PBS and then harvested with trypsin-EDTA and then centrifuged at 1200 rpm for 5 minutes. After the removal of the supernatant, ice-cold reagents were added to the cell pellet with a volume ratio of CER I:CER II:NER at 200 : 11 : 100. After a high-speed vortex for 15 seconds to fully suspend, the reactions were incubated on ice for 10 minutes and then ice-cold CER II were added, followed by a 5-second vortex and 1 minute incubation. After a 5-minute centrifugation at the maximum speed (15000 rpm), resultant supernatant (cytoplasmic extract) was transferred to a clean pre-chilled tube and store at −80°C. Meanwhile, the insoluble (pellet) fraction was resuspended in ice-cold NER by vortexing for 15 seconds. This vortex procedure was repeated for 4 times with a 10-minute incubation on ice between each vortex. A centrifugation at the maximum speed (15000 rpm) was conducted after the final vortex and the resultant supernatant (nuclear extract fraction) was transferred to a clean tube and stored at −80°C.

### 2.5. Western Blot Analysis

Cell lysates were separated in a 10% or 15% polyacrylamide gel and transferred onto a PVDF membrane (Millipore Corporation, Milford, MA, USA). The blot was subsequently incubated with 3% nonfat milk in PBS for 1 hour to block nonspecific binding and probed with a corresponding antibody against a specific protein for 37°C at 2 hours or overnight at 4°C, and then with an appropriate peroxidase conjugated secondary antibody for 1 hour. Extensive washing with wash buffer was conducted between each incubation and after the final washing, signal was developed by ECL detection system and relative photographic density was quantitated by a gel documentation and analysis system (Alpha Imager 2000, Alpha Innotech Corporation).

### 2.6. Cell Transfection

Cells were grown on 6-well cell culture dish overnight and then transfected with 4 *μ*g of pEGFPC1-LC3 [21] for 6 hours, followed by an indicated treatment. Afterwards, cells were fixed with 2% paraformaldehyde for 12 minutes and then incubated with 0.5% Triton X-100 for 10 minutes. Extensive PBS washing was conducted between each reaction to remove any residual solvent. The dot formation of GFP-LC3 was detected under a fluorescence microscope after drug treatment.

### 2.7. DAPI Staining

4 × 10^5^ cells were grown on 6-well cell culture dish overnight and followed by drug treatment. After drug treatment, cells were fixed with 4% paraformaldehyde for 12 minutes. Extensive PBS washing was conducted between each reaction to remove any residual solvent. Cells were subjected to DAPI staining for 5 minutes and then observed under fluorescence microscopy equipped with filters for UV.

### 2.8. Mitochondrial Membrane Potential Assay

To measure mitochondrial membrane potential, Huh7 cells were seeded into 6-well cell culture dish containing growth medium at a density of 2 × 10^5^ cells per dish and treated with dioscin for 24 hours. After the incubation, cells were washed and stained with 5 *μ*g/mL JC-1. The mitochondrial membrane potential collapses; the monomeric JC-1 remains cytosolic and stains the cytosol with a green color in apoptotic cells. On the other hand, in nonapoptotic cells, JC-1 impulsively forms complexes and aggregates with intense red fluorescence. The loss of mitochondrial membrane potential was observed under fluorescence microscopy equipped with filters for Blue 488 nm and Green 543 nm. 

### 2.9. Annexin V/PI Double Staining

To detect apoptosis in Huh7 cells after exposure to dioscin, an FITC Annexin V Apoptosis Detection Kit I was used to quantify cell numbers in different stages of cell death [22]. Briefly, 1 × 10^5^ cells were resuspended in 100 *μ*L 1x binding buffer (0.01 M Hepes/NaOH (pH 7.4), 0.14 M NaCl, 2.5 mM CaCl_2_). With an addition of 5 *μ*L of FITC Annexin V and 5 *μ*L PI, the cell suspension was gently mixed and then incubated for 15 minutes at room temperature in the dark. Afterwards, 400 *μ*L of 1x binding buffer was added to each tube followed by flow cytometry analysis within 1 hour. 

### 2.10. Statistical Analysis

Statistical significance of differences throughout this study was analyzed by One-way ANOVA test. A *P* value <0.05 was considered to be statistically significant. Values represent the means ± standard deviation, and the experiments were repeated three times. 

## 3. Results

### 3.1. Dioscin Induced Cell Death via Apoptosis in Huh7 Cells

 To assess the effects of dioscin on cell viability, Huh7 cells were treated with dioscin and then analyzed with MTT assay and cell count assay. As shown in Figures 1(b) and 1(c), after a treatment with dioscin of various concentrations for 24 hours, cell viability was significantly reduced in a dose-dependent manner, as compared with that of untreated cells. Results from MTT assay showed that around 40% of cells survived after a treatment of dioscin at 5 *μ*M. Since trypan blue staining indicating cell death, microscopic cell counting revealed a dramatic decrease in viable cell numbers in dioscin-treated Huh7 cells compared to that of control untreated cells.

### 3.2. Induction of Apoptosis Is Dependent on the Activation of Caspase-3 and Caspase-9 in Dioscin-Treated Huh7 Cell Lines

**Figure 2:**
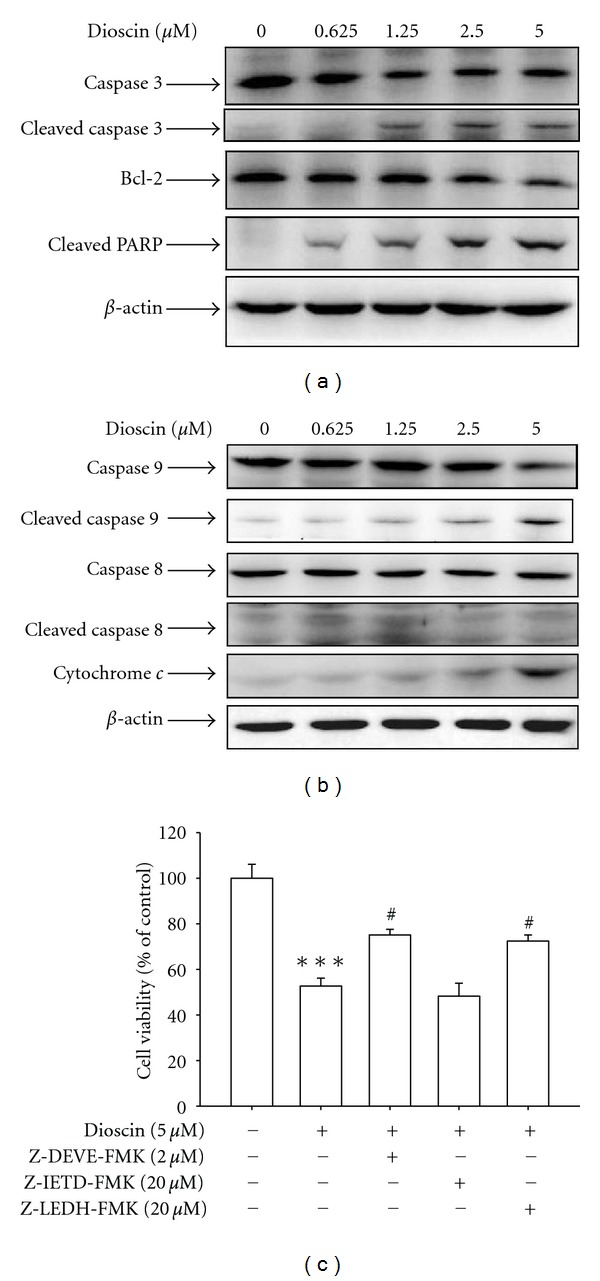
Dioscin may induce the activation of caspases in Huh7 cells. Cells were treated with an indicated concentration of dioscin for 24 hours and then analyzed by western blotting with an antibody against Bcl-2, PARP, or caspase-3 (a). Meanwhile, the expression of cleaved caspase-8, -9, and cytochrome c release was also analyzed with that of *β*-actin as an internal control (b). Furthermore, cells were treated with 5 *μ*M dioscin for 24 hours in the presence or absence of 2 *μ*M Z-DEVE-FMK, 20 *μ*M Z-IETD-FMK, and 20 *μ*M Z-LEHD-FMK and then subjected to MTT assay for cell viability (c). Results are shown as mean ± SD from 3 determinations per condition repeated 3 times. ***P < 0.001, control versus dioscin; ^#^ *P* < 0.05, dioscin versus Z-DEVE-FMK, Z-IETD-FMK, and Z-LEHD-FMK plus dioscin.

 To determine whether dioscin-induced cell death is related to apoptosis, DAPI staining was performed to analyze the changes in nuclear morphology. Results revealed the condensed and fragmented nuclei at a concentration of 2.5 *μ*M or higher of dioscin (Figure 1(d)). Further, mitochondrial membrane potentials in dioscin-treated Huh7 cells were measured to discover that the mitochondrial membrane potential of Huh7 cells was decreased by dioscin treatment (Figure 1(e)). Annexin V/PI double staining was also determined by flow cytometry, and results showed an increased percentage of cells displaying phosphatidyl serine (PS) externalization in dioscin-treated Huh7 cells (Figure 1(f)). To investigate underlying mechanisms involved in dioscin-induced apoptosis, apoptosis-related molecules were examined by western blotting. Results indicated that after a 24-hour treatment of dioscin, levels of cytochrome c release, cleaved PARP, and activated caspase 3 and 9 were increased, while activated caspase-8 remained unchanged. On the other hand, decreased expression level of Bcl-2, an antiapoptosis protein, was also detected in Huh7 cells treated with dioscin for 24 hours (Figures 2(a) and 2(b)). To further confirm the involvement of caspase activation in dioscin-induced apoptosis, caspase-specific inhibitors were used. Results shown in Figure 2(c) indicated that apretreatment with caspase-3- and -9-specific inhibitors both could effectively attenuate dioscin-induced cell apoptosis (Figure 2(c)). These data suggest that dioscin-induced apoptosis is dependent on the activation of caspase-3 and caspase-9, but not that of caspase-8.

### 3.3. Dioscin-Induced Apoptosis Was Dependent on ERK1/2 Activation and Subsequent Caspase-3/-9 Pathway

**Figure 3:**
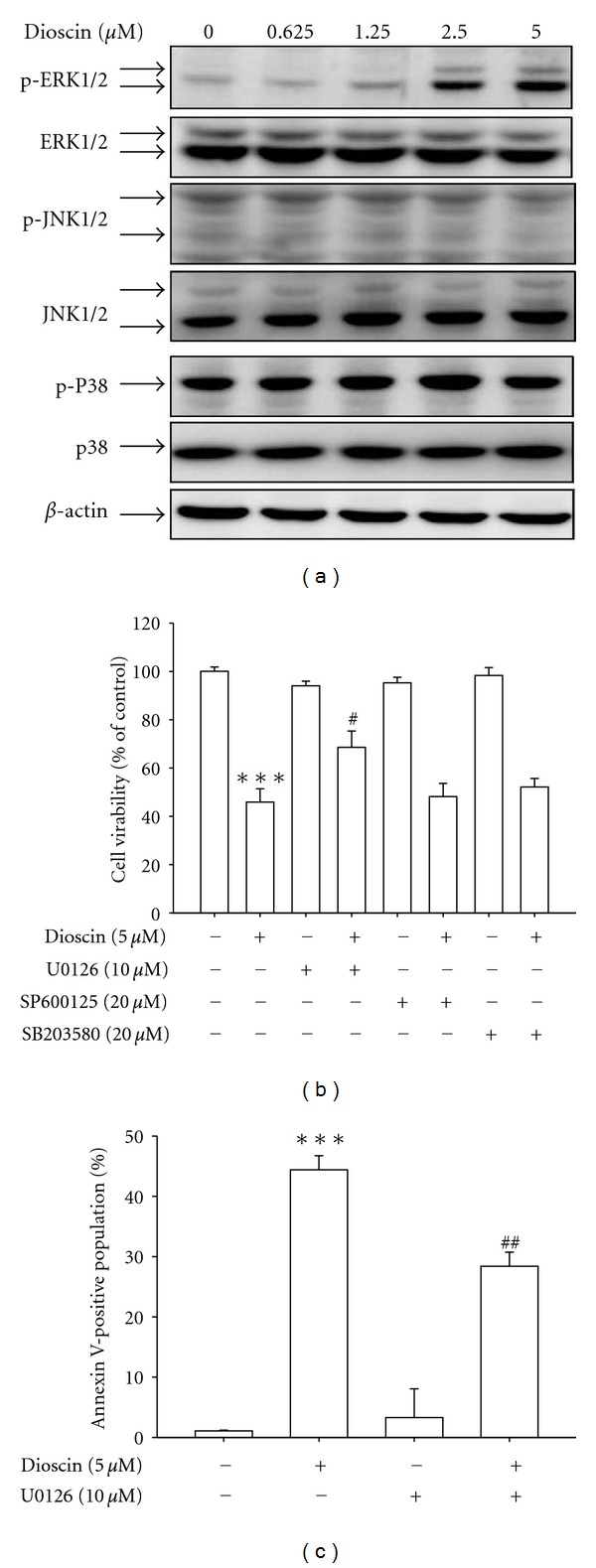
Dioscin may enhance the activation of MAPK in Huh7 cells. Cells were treated with an indicated concentration of dioscin for 24 hours and then analyzed by western blotting with an appropriate antibody to investigate the expression of phosphorylation of ERK1/2, JNK1/2, and p38 with *β*-actin acting as an internal control (a). Cells were treated with 5 *μ*M dioscin for 24 hours in the presence or absence of 10 *μ*M U0126, 20 *μ*M SB203580, and 20 *μ*M SP600125 and then subjected to MTT assay for cell viability. Results are shown as mean ± SD from 3 determinations per condition repeated 3 times (b). Huh7 cells were treated with 5 *μ*M dioscin for 24 hours in the presence or absence of 10 *μ*M U0126 and then subjected to Annexin-V and PI double-stained flow cytometry (c). Results are shown as mean ± SD. ***P < 0.001, control versus dioscin; ^#^ *P* < 0.05, dioscin versus MAPK inhibitor plus dioscin; ^##^ *P* < 0.01, dioscin versus U0126 plus dioscin.

 Previous studies reported that mitogen-activated protein kinase (MAPK) family played as a multifunctional mediator of signal transduction processes, including cell death, differentiation, proliferation, and migration [23, 24]. To investigate the possible role of MAPK pathways in dioscin-induced apoptosis, the expression levels of the phosphorylated forms of ERK1/2, p38MAPK, and JNK1/2 were examined by western blotting. Results shown that dioscin treatment may lead to an increase in the activation of ERK1/2 in a dose-dependent manner (Figure 3(a)). Furthermore, cells were subjected to a pretreatment with MAPK-specific inhibitors followed by a 24-hour treatment of 5 *μ*M dioscin. Results from MTT assay and Annexin V/PI double staining showed that dioscin-induced apoptosis was attenuated by inhibiting ERK1/2 activation (Figures 3(b) and 3(c)). 

### 3.4. Induction of Autophagy in Dioscin-Treated Huh7 Cell Lines

**Figure 4:**
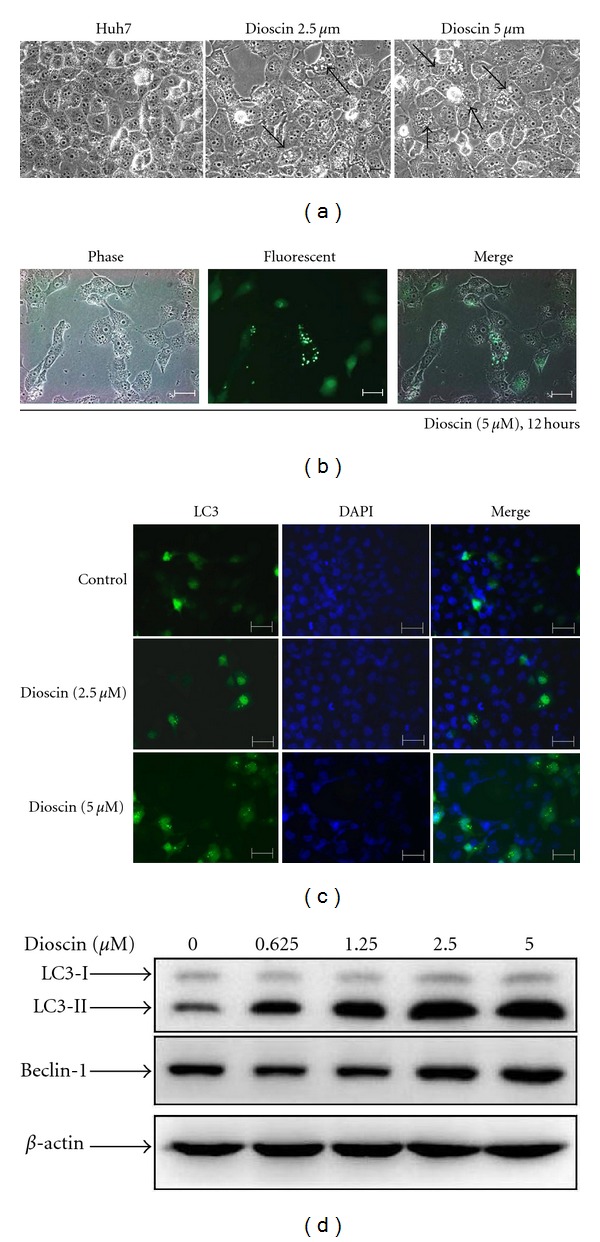
Autophagy was induced in dioscin-treated cells. Huh7 cells were treated with 2.5 and 5 *μ*M dioscin for 12 hours and a microscopic observation revealed the formation vacuoles in cytoplasms of treated cells (a). After a successful transfection with pEGFPC1-LC3, Huh7 cells were treated with dioscin of 5 *μ*M for 12 hours, followed by an observation for LC-3 (green fluorescence) under fluorescence microscopy (b). After a 24-hour treatment with dioscin, DAPI staining and subsequent fluorescence microscopy for LC-3 and DAPI (blue fluorescence) were conducted (c), as well as western blotting with antibodies against LC3 and beclin-1 (d). Scale bars = 100 *μ*M.

 Previous data showed that dioscin treated cells displayed characteristic apoptotic changes in cell morphology. Moreover, various numbers of vacuoles were observed in the cytoplasm at 12 hours after dioscin treatment (Figure 4(a)). The formation of vacuoles in dioscin-treated cells are similar to that in cell autophagy [25], a general phenomenon that occurs when cells response to stress. To determine whether dioscin also induces autophagy, LC3-II protein and Beclin-1, two autophagy-related proteins, were analyzed. As shown in Figures 4(b) and 4(c), after a transfection of pEGFPC1-LC3 and a treatment of dioscin for 12 and 24 hours, cytoplasmic LC3 formation was observed, which indicated the formation of autophagosomes, in cells treated with dioscin. A significant change of LC3 puncta formation was found as soon as 12 hours after a treatment of dioscin 2.5 *μ*M while an increased LC3-II protein expression was also observed in at 24 hours at a dose-dependent manner. Compared to that of control, the protein expression of Beclin-1 was upregulated by dioscin (Figure 4(d)). 

### 3.5. Dioscin-Induced Cell Death Was Enhanced by the Treatment of Autophagy Inhibitors

**Figure 5:**
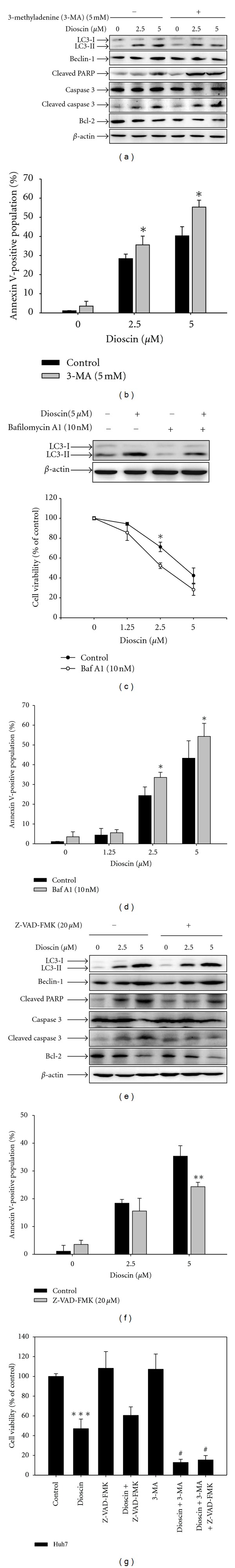
Autophagy inhibitors have effects on dioscin-induced cell death. Cells were treated with dioscin of 2.5 and 5 *μ*M for 24 hours in the presence or absence of autophagy inhibitor 3-MA of 5 mM and then subjected to western blotting for LC3-II, beclin-1, cleaved PARP, caspase 3, Bcl-2, and *β*-actin (a). Subsequently, these treated cells were double stained with Annexin-V and PI and then analyzed by flow cytometry (b). For another inhibitor BafA1, cells were treated with an indicated concentration of dioscin for 24 hours in the presence or absence of 10 nM BafA1 and then subjected to MTT assay for cell viability. The expression of LC3-II formation was investigated by western blotting with *β*-actin being an internal control (c). These treated cells were double-stained with Annexin-V and PI and subsequently analyzed by flow cytometry (d). Results are shown as mean ± SD. *P < 0.05, dioscin versus BafA1 plus dioscin. Cells were treated with dioscin of 2.5 and 5 *μ*M for 24 hours in the presence or absence of caspase inhibitor Z-VAD-FMK of 20 *μ*M and then subjected to western blotting for LC3-II, beclin-1, PARP, caspase 3, Bcl-2, and *β*-actin (e). Subsequently, these treated cells were double stained with Annexin-V and PI and then analyzed by flow cytometry (f). Furthermore, cells were treated with various combinations for 24 hours, including 20 *μ*M Z-VAD-FMK, 5 *μ*M dioscin plus 20 *μ*M Z-VAD-FMK, 5 mM 3-MA, 5 *μ*M dioscin plus 5 mM 3-MA, 5 *μ*M dioscin plus 20 *μ*M Z-VAD-FMK and 5 mM 3-MA, and then subjected to MTT assay for cell viability (g). Results are shown as mean ± SD (g). ***P < 0.001, control versus dioscin; ^#^ *P* < 0.05, dioscin versus 3-MA plus dioscin or Z-VAD-FMK and 3-MA plus dioscin.

 In order to clarify the interaction between dioscin-induced apoptosis and autophagy, two autophagy inhibitors acting at different stages, 3-MA and bafilomycin A1 (BafA1), were used in the following experiments. 3-MA, an autophagy inhibitor that can block autophagosome formation via the inhibition of type III PI-3K, was used. Huh7 cells were pretreated with 5 mM 3-MA for 1 hour and then dioscin for 24 hours and then subjected to western blotting. Results as for Figure 5(a) indicated that 3-MA pretreatment decreased the protein levels of dioscin-induced LC3-II and Beclin-1 and enhanced the expression levels of cleaved PARP and cleaved caspase-3. Meanwhile, the percentage of annexin V-positive cells was also higher in cells pretreated with 3-MA (Figure 5(b)). As for BafA1, an inhibitor of vacuolar ATPase to prevent the fusion between lysosomes and autophagosomes, cells were pretreated with BafA1 for 1 hour and then dioscin for 24 hours. As shown in Figure 5(c), cells pretreated with BafA1 were more susceptible to dioscin together with an increased percentage of annexin V-positivity (Figure 5(d)). 

 Furthermore, a cotreatment of dioscin and Z-VAD-FMK, a broad-spectrum caspase inhibitor, was conducted to show that dioscin-induced increase of cleaved PARP and cleaved caspase-3, as well as the percentage of annexin V-positivity, was abolished by Z-VAD-FMK, but dioscin-induced LC3-II and Beclin-1 expression remained unaltered (Figures 5(e) and 5(f)). A similar result was obtained in the MTT assay (Figure 5(g)). Clearly, inhibition of autophagy did not hinder dioscin-induced cell death, even further enhanced the cell toxicity of dioscin. Therefore, these results indicate that autophagy had a cytoprotective effect in dioscin-induced Huh7 cell death.

## 4. Discussion

 Natural herbal products provide one of the most important sources for the development of novel chemotherapeutics, which have been practiced traditionally in various ethnic societies worldwide. Extensive studies indicate that these herbal may arrest the tumor promotion and progression in various human cancer cell lines by controlling cell proliferation, invasion, or apoptosis. Our results show that dioscin may induce death of hepatic cancer cells. While dioscin-treated Huh7 cells showed significant changes in nuclei condense and mitochondrial membrane potential, the amounts of cleaved caspase-3, -9, and PARP were increased after treatment with dioscin together with a decreased expression of antiapoptotic proteins Bcl-2. These results indicated that dioscin may induce apoptosis of Huh7 cells through an activation of ERK1/2 signal pathway. The findings are in agreement with quercetin-induced apoptosis in A549 cells, and similar to the effects of saponins of 20-O-(beta-D-glucopyranosyl)-20(S)-protopanaxadiol (IH-901) [26] on ERK1/2 and subsequently cell death.

 Autophagy is an important cellular response for various environmental stimuli, diseases, and even cancers [27–29]. Many anticancer agents, including tamoxifen, rapamycin, arsenic trioxide, and temozolomide were reported to induce autophagy [30]. Further investigation revealed that sulforaphane causes autophagy as a defense mechanism against apoptosis in PC3 and LNCaP prostate cancer cells [31] and 7,7′′-Dimethoxyagastisflavone (DMGF) induced autophagic cell death in HepG2 cells [32]. In this study, dioscin resulted in apparent apoptosis at 24 hours, autophagy was observed as soon as 12 hours after dioscin was added to the culture medium (Figures 4(a) and 4(b)), and the expression of LC3-II indicated that the induction of autophagy was dose-dependent (Figure 4(d)). 

 Previous studies have suggested that autophagy can be induced by various compounds and involved in cell death or cytoprotection in HCC cell lines [17, 32, 33]. To further investigate the role of autophagy in dioscin-induced cell death, an autophagy inhibitor, 3-MA was used. Results showed that 3-MA it could inhibit the maturation of LC3-II, which did not lead to a decrease, but rather increase, in dioscin-induced cell death. A previous study has reported that inhibition of autophagy at different stages has opposite effects on cell survival [34], and in this study, inhibition of autophagy leads to enhanced apoptosis at early stages. Treatment with caspase inhibitor alone may restore a small percentage of cell viability. Neither inhibitor showed any protective effect suggesting that autophagy is an important mechanism in dioscin-induced apoptosis in Huh7 cell lines. Autophagy also serves as a critical defensive mechanism against common chemotherapeutic agents.

 Autophagy is suppressed by functional p53 and certain cytoplasmic p53 mutants [35] and a previous study pointed out that LC3-II formation was not detected in DU145 cells treated with Zoledronic Acid [36]. Tasdemir et al. suggest that cytoplasmic p53 suppresses autophagy and p53 inhibition induces autophagy [37]. Furthermore, nuclear expression of p53 may stimulate autophagy by DRAM upregulation and mTOR inhibition [38, 39]. This suggests that p53 has the opposite effect on autophagy regulation. Therefore, dioscin-induced autophagy and apoptosis in Huh7 cells may be partially attributed to the lack of functional p53, which could be verified by additional experiments exploring the role of p53 in dioscin-induced autophagy. Furthermore, more experiments conducted in other types of cancer cells may provide more data to identify signaling pathways involved in dioscin-induced autophagy. Autophagic agonists have been described as anticancer drugs [32, 40, 41] with various compounds being shown to induce death in tumor cells with defective apoptotic machinery [42, 43]. Thus, with the capability to induce both the apoptotic and autophagic pathways, dioscin may be a good candidate for antitumor treatment.

 In conclusion, this study is the first to demonstrate that dioscin suppressed cell growth and induced apoptosis in Huh7 cells through the activation of ERK1/2 signal pathway. By inhibiting cell growth and inducing autophagy in the early stage of dioscin-induced apoptosis, the anticancer properties of dioscin are quite promising. We also found that combination of dioscin with autophagy inhibitors may strengthen the efficiency of proapoptotic chemotherapeutic strategies, suggesting that autophagy protects cancer cells from the anticancer activity of dioscin in Huh7 cells.
