# Corrigendum to “Autophagy Inhibition Enhances Apoptosis Induced by Dioscin in Huh7 Cells”

**DOI:** 10.1155/2017/7143563

**Published:** 2017-11-21

**Authors:** Ming-Ju Hsieh, Shun-Fa Yang, Yih-Shou Hsieh, Tzy-Yen Chen, Hui-Ling Chiou

**Affiliations:** ^1^School of Medical Laboratory and Biotechnology, Chung Shan Medical University, 110, Section 1, Chien-Kuo N. Road, Taichung 402, Taiwan; ^2^Institute of Medicine, Chung Shan Medical University, 110, Section 1, Chien-Kuo N. Road, Taichung 402, Taiwan; ^3^Department of Medical Research, Chung Shan Medical University Hospital, 110, Section 1, Chien-Kuo N. Road, Taichung 402, Taiwan; ^4^Department of Biochemistry and Institute of Biochemistry and Biotechnology, Chung Shan Medical University, 110, Section 1, Chien-Kuo N. Road, Taichung 402, Taiwan; ^5^Department of Internal Medicine, Chung Shan Medical University Hospital, 110, Section 1, Chien-Kuo N. Road, Taichung 402, Taiwan; ^6^Department of Internal Medicine, School of Medicine, Chung Shan Medical University, 110, Section 1, Chien-Kuo N. Road, Taichung 402, Taiwan; ^7^Department of Clinical Laboratory, Chung Shan Medical University Hospital, 110, Section 1, Chien-Kuo N. Road, Taichung 402, Taiwan

In the article titled “Autophagy Inhibition Enhances Apoptosis Induced by Dioscin in Huh7 Cells” [1], there was an error in Figures 2(a) and 2(b). The authors provided the western blots for caspase-8 and actin for the figures in triplicate. Therefore, Figure 2 should be corrected as follows.

## Figures and Tables

**Figure 2:**
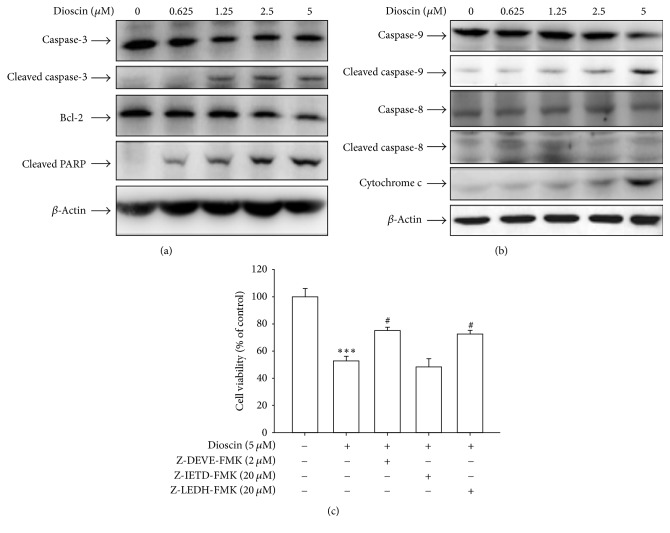
Dioscin may induce the activation of caspases in Huh7 cells. Cells were treated with an indicated concentration of dioscin for 24 hours and then analyzed by western blotting with an antibody against Bcl-2, PARP, or caspase-3 (a). Meanwhile, the expression of cleaved caspase-8, caspase-9, and cytochrome c release was also analyzed with that of *β*-actin as an internal control (b). Furthermore, cells were treated with 5 *μ*M dioscin for 24 hours in the presence or absence of 2 *μ*M Z-DEVE-FMK, 20 *μ*M Z-IETD-FMK, and 20 *μ*M Z-LEHD-FMK and then subjected to MTT assay for cell viability (c). Results are shown as mean ± SD from 3 determinations per condition repeated 3 times. ^*∗∗∗*^*P* < 0.001, control versus dioscin; ^#^*P* < 0.05, dioscin versus Z-DEVE-FMK, Z-IETD-FMK, and Z-LEHD-FMK plus dioscin.
